# Synthesis and applications of cyclonucleosides: an update (2010–2023)

**DOI:** 10.1007/s11030-023-10740-5

**Published:** 2023-10-27

**Authors:** Katherine Burchiellaro, Adam Mieczkowski

**Affiliations:** 1 Institute of Biochemistry and Biophysics, Polish Academy of Sciences, Pawinskiego 5a, 02-106 Warsaw, Poland; 2 Faculty of Chemistry, University of Warsaw, Pasteura 1, 02-093 Warsaw, Poland

**Keywords:** Nucleosides, Cyclonucleosides, Purines, Pyrimidines, Bicyclic, Inhibitors, Biocides, Heterocycles

## Abstract

Cyclonucleosides are a group of nucleoside derivatives which, in addition to the classical *N*-glycosidic bond, have an additional covalent bond (linker, bridge) in their structure, which connects the heterocyclic base and sugar ring. The majority of them have been discovered in the laboratory; however, few such compounds have also been found in natural sources, including metabolites of sponges or radical damage occurring in nucleic acids. Due to their structural properties—rigid, fixed conformation—they have found wide applications in medicinal chemistry and biochemistry as biocides as well as enzyme inhibitors and molecular probes. They have also found use as convenient synthetic tools for the preparation of new nucleoside analogues, enabling structural modifications of both the sugar ring and heterocyclic base. This review summarizes the recent progress in the synthesis of various purine and pyrimidine cyclonucleosides using diverse chemical approaches based on radical, “click”, metal-mediated, and other types of reactions. It also presents recent reports concerning possible applications in medicinal chemistry, as well as their applications as valuable key intermediates in the synthesis of sugar- and base-modified nucleoside analogues and heterocyclic compounds.

**Graphical abstract:**

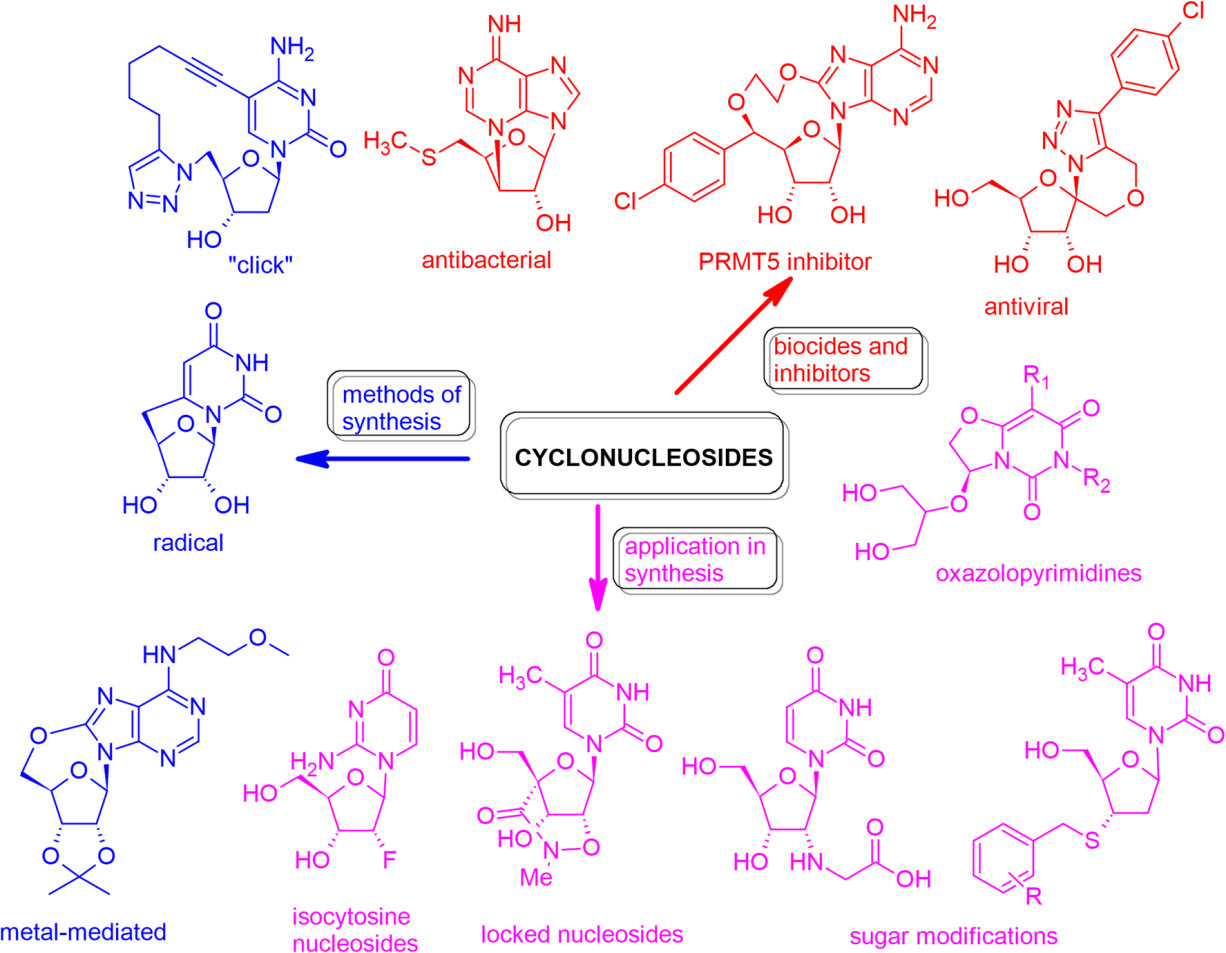

## Introduction

Cyclonucleosides are nucleoside derivatives and analogues, which except for typical *N*-glycosidic bonds, have additional covalent linkages, that connect the sugar ring and heterocyclic base. Such compounds have a rigid structure and fixed conformation [1, 2], which determines their physicochemical and biochemical properties. Cyclonucleosides have so far been used in medicinal chemistry to obtain the prodrug of the antileukemic drug Cytarabine (Ancitabine) [3], inhibitors of nucleoside-related enzymes such as uridine phosphorylase *UrdPase* (enhancing the effect of anticancer drugs) [4], antiviral drugs [5] and molecular probes to investigate enzymatic reactions, such as hydrolysis with ribonuclease A (*RNase-A*) [6]. These compounds could also be used in organic chemistry as valuable intermediates in the synthesis of further nucleoside analogues and other heterocyclic systems [1]. Purine 8,5′-cyclonucleoside lesions are tandem-type lesions observed in the DNA structure, formed under the free radical stress caused by the attack of HO• radicals to 2-deoxyribose units followed by the formation of 8,5′-linkage. Such DNA modifications identified in mammalian cellular DNA in vivo are deeply studied for their potential involvement in human health [7]. It is also believed that pyrimidine cyclonucleosides may have played an important role in the prebiotic environment, being involved in the spontaneous synthesis of nucleic acid components [8–10]. Although most of the cyclonucleosides were designed and synthesized in the laboratory, purine cyclonucleoside such as *N*^3^,5′-cycloxanthosine or its uric acid analogue were isolated from natural sources from *Eryus* [11] and *Axinella* sponges [12].

The methods of synthesis of cyclonucleosides and their applications in medicinal chemistry were described in two review articles published by the corresponding author 12 years ago [13, 14]. Some aspects of cyclonucleoside chemistry were also included in two consequent reviews published in 2012 [15] and 2013 year [16]. Since then, many new articles about cyclonucleosides have appeared, revealing new approaches to synthesis, as well as their new applications in organic synthesis and for the invention and development of biologically active substances. For this reason, we saw the need and opportunity to collect new results and achievements obtained in research on this group of nucleoside analogues. In the first part of our article, we would like to focus on the synthetic paths and biochemical properties of novel pyrimidine and purine cyclonucleosides, while in the second part, we would like to present various applications of cyclonucleosides in the synthesis of the other nucleoside analogues and heterocyclic compounds.

## The synthesis of cyclonucleosides

### The synthesis of pyrimidine cyclonucleosides

**Scheme 1:**
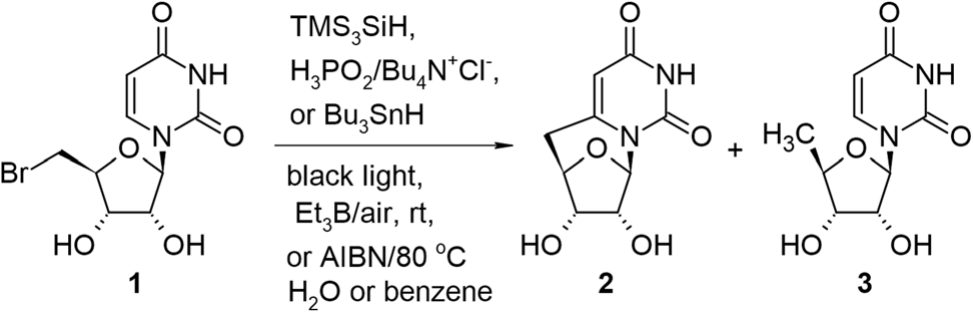
The synthesis of *C*-bridged 6,5′-cyclo-2′,3′-dideoxyuridine (**2**) under radical conditions

The free radical stress conditions are mainly responsible for cyclonucleoside-containing lesions observed in the DNA structure within living cells [7]. Similarly, radical reactions are convenient approaches for the formation of cyclonucleosides in the chemical laboratory. Perchyonok reported the black light-induced radical cyclization approach to pyrimidine 6,5′-cyclonucleosides testing various free radical hydrogen donors [17]. The author studied tandem radical 1,6-HAT-cyclization of 5′-bromo-5′-deoxyuridine (**1**) in the presence of such hydrogen donors as TMS_3_SiH, H_3_PO_2_/Bu_4_N^+^Cl^−^, Bu_3_SnH, using water or benzene as solvents, in the presence of such radical initiators as black light, Et_3_B/air in room temperature and AIBN in 80 °C (Scheme 1). It was observed in all cases, that intramolecular radical 6-*exo*-trig reaction leading to *C*-bridged 6,5′-cyclo-2′,3′-dideoxyuridine (**2**) was the main reaction mechanism in the examined process and intermolecular hydrogen transfer reaction resulted in 5′-deoxyuridine (**3**) is much slower when compared to intramolecular radical 6-*exo*-trig cascade reaction.

**Scheme 2:**
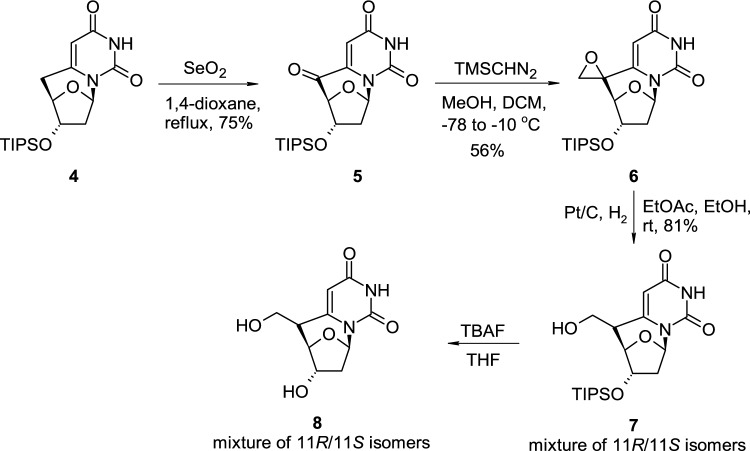
Bridge modifications of *C*-bridged 6,5′-cyclo-2′,5′-dideoxyuridine **4** through oxidation-epoxide formation-epoxide hydrogenation protocol

The methylene bridge formed in the intramolecular radical 6-*exo*-trig cyclisation could be further subjected to chemical modifications (Scheme 2) [18]. Triisopropylsilyl-protected (TIPS-protected) 6,5′-cyclo-2′,5′-dideoxyuridine (**4**) was treated with SeO_2_ in boiling dioxane, which resulted in the oxidation of C′5 position and introduction of the carbonyl group to the methylene bridge. Intermediate **5** was transformed into spiro-oxirane derivative **6** using trimethylsilyldiazomethane in a mixture of MeOH and DCM and subsequent hydrogenation on Pd/C led to the partially protected 5′-(hydroxymethyl)-6,5′-cyclo-2′,5′-dideoxyuridine (**7**) obtained as a mixture of two isomers. TIPS protecting group in cyclonucleoside **7** was removed using TBAF (tetra-*n*-butylammonium fluoride) in THF, and final product **8** was separated into two isomers using HPLC. The authors also reported, that pure (*R*)-8 and (*S*)-8 isomers readily undergo epimerization reaction and interconversion under the aqueous basic conditions.

**Scheme 3:**
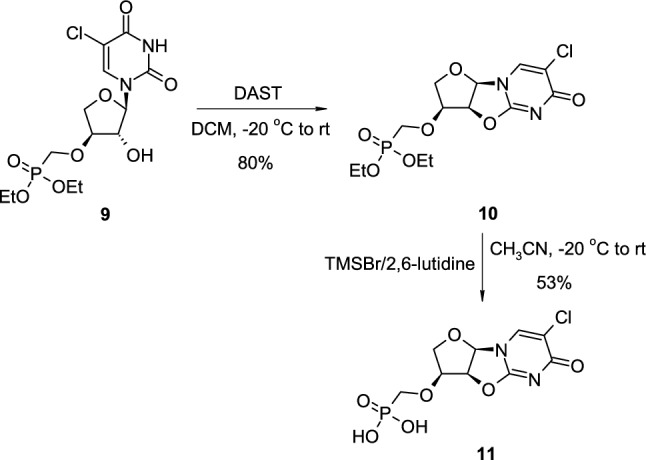
The synthesis of 2,2′-*O*-cyclo-L-threonucleoside 3′-*O*-phosphonic acid **11** using DAST as cyclisation agent

Zhu reported that the synthesis of 2,2′-*O*-cyclouridine analogues could be easily performed using diethylaminosulfur trifluoride (DAST) as cyclisation agent [19]. In the representative example (Scheme 3), pyrimidine L-threonucleoside phosphonate ester **9** was treated with DAST in dichloromethane at - 20 °C which resulted in corresponding 2,2′-*O*-cyclo-L-threonucleoside phosphonate ester **10**. Deprotection of phosphonate ester to free phosphonic acid smoothly underwent by treatment with TMSBr/2,6-lutidine leading to 2,2′-*O*-anhydro-L-threonucleoside 3′-*O*-phosphonic acid **11**.

**Scheme 4:**
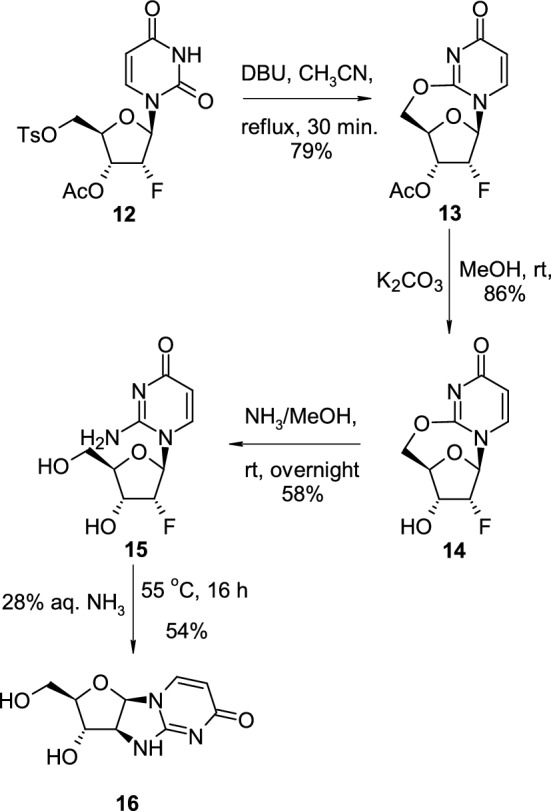
The synthesis of 2′-deoxy-2′-fluoro-2,5′-*O*-cyclouridine (**14**) and its conversion to 2,2′-imino-1-(2-deoxy-β-D-arabinofuranosyl)uracil (**16**)

Ingale reported the synthesis of 2,5′-*O*-cyclouridine nucleoside and its transformation into novel 2,2′-imino cyclonucleoside through intermediate isocytosine riboside [20] (Scheme 4). 3′-*O*-acetyl-5′-*O*-tosyl-2′-deoxy-2′-fluorouridine (**12**), when treated with 1,8-diazabicyclo[5.4.0]undec-7-ene (DBU) in boiling acetonitrile, was transformed into acetyl-protected 2′-deoxy-2′-fluoro-2,5′-*O*-cyclouridine nucleoside **13**. Removal of acetyl-protected group with potassium carbonate, and treatment deprotected 2′-deoxy-2′-fluoro-2,5′-*O*-cyclouridine (**14**) with methanolic ammonia led to the isocytosine 2′-deoxy-2′-fluororiboside (**15**). Nucleoside **15**, when treated with aqueous ammonia solution at elevated temperatures, underwent cyclisation leading to another type of cyclonucleoside, 2,2′-imino-1-(2-deoxy-β-D-arabinofuranosyl)uracil (**16**), possessing an imino group in the linkage connecting ribose and pyrimidine rings.

**Scheme 5:**
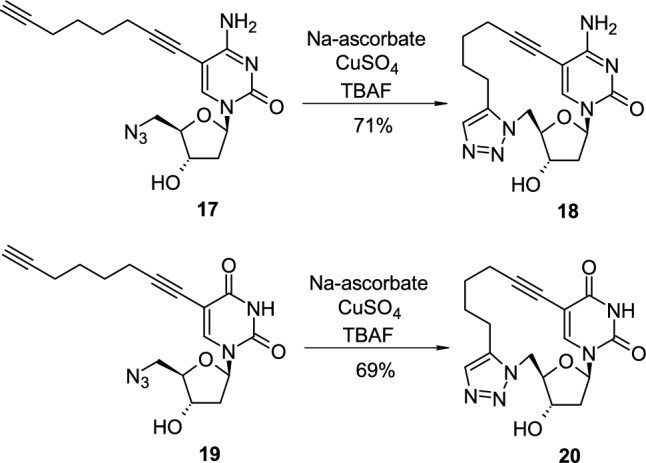
The synthesis of 5,5′-triazole-linked pyrimidine cyclonucleosides **18**,**20** obtained through Cu(I)-promoted 1,3-dipolar intramolecular cycloaddition of azide and alkyne

Copper(I)-promoted “click” 1,3-dipolar intramolecular cycloaddition of azide and alkyne could be a convenient method for cyclonucleoside synthesis. Liu and co-workers reported the synthesis of a modified 2′-deoxycytidine **17** bearing azide group at the C'5 position and octa-1,7-diyne group at the C5 position (Scheme 5) [21]. Compound **17** was subjected to azide ‘click’cycloaddition reaction in the presence of sodium ascorbate, copper sulphate and TBAF, which resulted in the formation of cyclonucleoside **18** with high yield. In a similar way, the modified 2′-deoxyuridine **19** bearing azide group at the C'5 position and octa-1,7-diyne group at the C5 position was also subjected to azide “click” cycloaddition reaction using the above conditions which led to the cyclonucleoside **20** (Scheme 5).

**Scheme 6.**
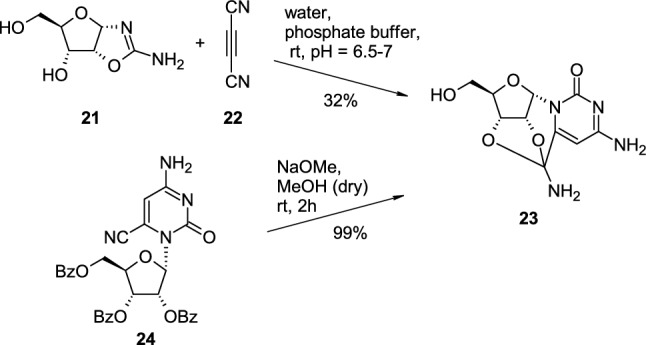
Two approaches to α-cytidine cyclonucleoside **23** from ribose aminooxazoline **21** or protected 6-cyano-α-cytidine **24**

During the investigation on the prebiotic synthesis of nucleoside derivatives, Tsanakopoulou reported the condensation between ribose aminooxazoline **21** with dicyanoacetylene (**22**), in a phosphate buffer at room temperature, which surprisingly led to the formation of α-cytidine cyclonucleoside **23** with 32% yield [22]. Compound **23** was also synthesized in 6 steps from α-cytidine, and the total yield of the whole process was also 32% (Scheme 6). The key step of second approach was based on basic hydrolysis and concomitant removal of benzyloxy groups of protected 6-cyano-α-cytidine **24** to the final product which was obtained with almost quantitative yield.

### The synthesis of purine cyclonucleosides

**Scheme 7:**
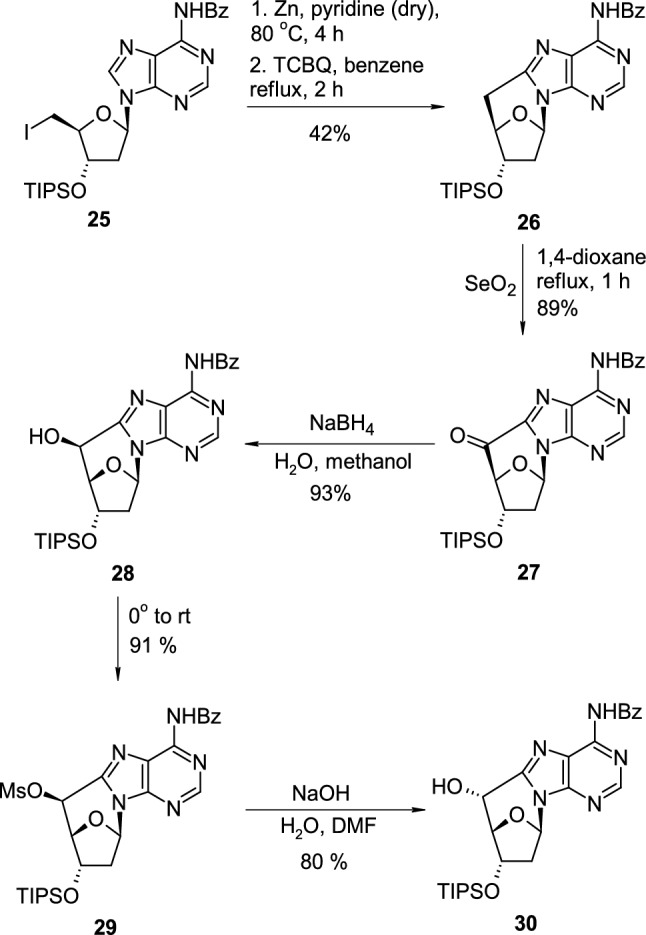
The synthesis and modifications of *C*-bridged 8,5′-cyclo-2′,5′-dideoxyadenosine nucleosides **26**–**30**

The radical cyclisation of 5′-deoxy-5′-halogen-substituted pyrimidine nucleosides, presented in Scheme 1, leading to the formation of a 6,5′-methylene bridge, could also be applied to purine nucleosides. Yueh reported [23] that protected 2′,5′-dideoxy-5′-iodoadenosine **25** heated with zinc powder in pyridine and treated with tetrachloro-1,4-benzoquinone (TCBQ) is transformed into protected 8,5′-cyclo-2′,5′-dideoxyadenosine **26** (Scheme 7). The methylene bridge in cyclonucleoside **26** could be further oxidized with SeO_4_ in boiling dioxane to give ketone **27**. The reduction of ketone to the hydroxy group resulted in alcohol **28** was performed with sodium borohydride in a methanol–water mixture. The authors also presented that protected 8,5′-(*S*)-cyclo-2′,5′-dideoxyadenosine (**28**) could be transformed into mesylate **29**, which underwent nucleophilic substitution after treatment with NaOH, leading to diastereomeric 8,5′-(*R*)-cyclo-2′,5′-dideoxyadenosine (**30**). Similar transformations were also applied to pyrimidine nucleosides. Obtained purine and pyrimidine cyclonucleosides were incorporated into DNA to study how the cyclo linkage affects the stability of duplex formation.

**Scheme 8:**
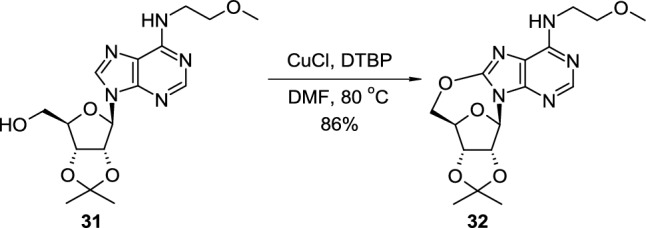
Cu-mediated synthesis of purine 8,5′‑*O*-cyclonucleoside **32** using DTBP as an oxidant

Yu reported copper-catalysed, radical intramolecular alkoxylation leading to purine 8,5′‑*O*-cyclonucleosides [24] (Scheme 8). Isopropylidene- or cyclohexylidene-protected purine nucleosides, such as 2′,3′-*O*-isopropylidene-*N*^6^-(2-methoxyethyl)adenosine (**31**), were heating in DMF in the presence of 0.5 equiv. CuCl and 3 equiv. of di-*tert*-butyl peroxide (DTBP, oxidant), which resulted in the formation of appropriate acetal protected 8,5′‑*O*-linked products such as 8,5′‑*O*-cycloadeosine derivative **32**, deprotected with trifluoroacetic acid–water-MeOH mixture (reflux, 5 h). The authors also observed that anchimeric assistance of the acetal protecting group was necessary for the efficient cyclisation process as deprotected nucleosides did not give the expected products.

**Scheme 9:**
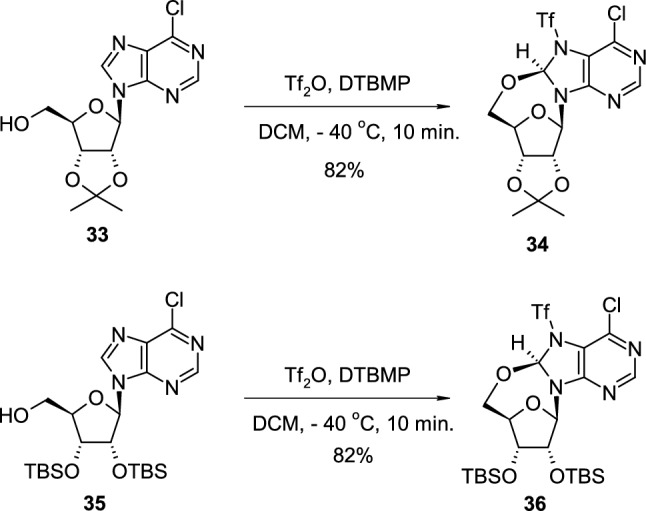
The synthesis of *N*7-triflate-substituted purine 8(*R*),5′-*O*-cycloribonucleosides **34**,**36** using Tf_2_O/DTBMP system

Amiable reported the unprecedented formation of 8(*R*),5′-*O*-cycloribonucleosides through a triflation reaction of purine ribonucleosides [25]. Isopropylidene-protected 6-chloropurine riboside **33** was subjected to a sulfonylation reaction with triflate anhydride in the presence of 2,6-di-*tert*-butyl-4-methylpyridine (DTBMP) (Scheme 9). Instead of the formation of the expected *O*-triflyl derivative, the authors observed the exclusive formation of cyclonucleoside **34** with a high yield, bearing the triflate group at *N*7 position of the purine ring. The triflation reaction of the purine ring instead of the 5′–OH group of the sugar ring, with concomitant formation of cyclonucleoside product, was also observed for 2′,3′-di(*tert*-butyldimethylsilyl)-6-chloropurine riboside (**35**). Nucleoside **35** treated with triflate anhydride in the presence of DTBMP was also transformed to appropriate cyclonucleoside **36** with a similar 82% yield.

**Scheme 10:**
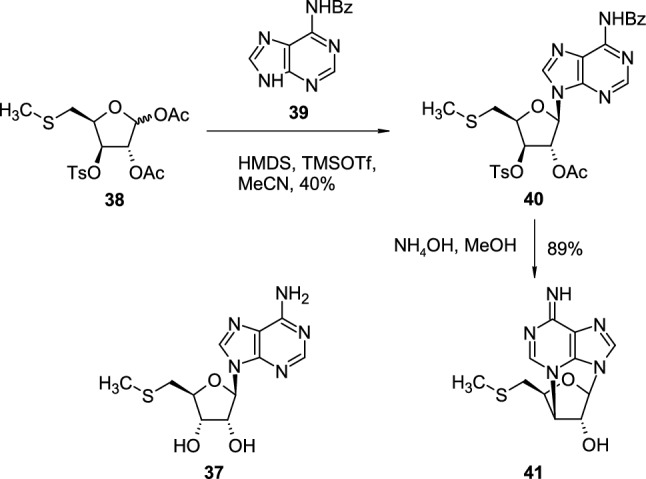
The synthesis of MTA cyclic analogue **41** in reaction of 3′-*O*-toluenesulphonyl adenosine derivative **40** with NH_3_ in methanol

5′-Methyl-thioadenosine/S-adenosylhomocysteine (MTA/AdoHcy) nucleosidase is an enzyme which cleaves the glycosidic bond in *S*-adenosylhomocysteine (AdoHcy, SAH) and 5′-methylthiodenosine (MTA, **37**) giving adenine and the corresponding thioribose (*S*-ribosylhomocysteine or 5′-methylthioribose respectively). Nucleoside analogues of MTA, acting as inhibitors of MTA/AdoHcy nucleosidase, could be used as antibacterial agents. De Carvalho reported the synthesis of cyclic MTA analogue as a potential antimicrobial nucleoside starting from protected 5′-methylthio sugar **38**, obtained in eight steps from D-glucose [26]. Acetyl-protected sugar **38** was coupled with *N*-benzoyladenine (**39**) in the presence of hexamethyldisilazane (HMDS) and trimethylsilyl triflate (TMSOTf) in acetonitrile, giving 3′-*O*-toluenesulphonyl adenosine derivative **40** (Scheme 10). The treatment of nucleoside **40** with ammonia in methanol led to the removal of protecting acetyl and benzoyl groups with concomitant formation of 2′,*N*^3^-cyclonucleoside **41**, which exhibits antibacterial activity but the low solubility of the compound in water made it impossible to determine the exact values of minimal inhibitory concentrations.

**Fig. 1:**
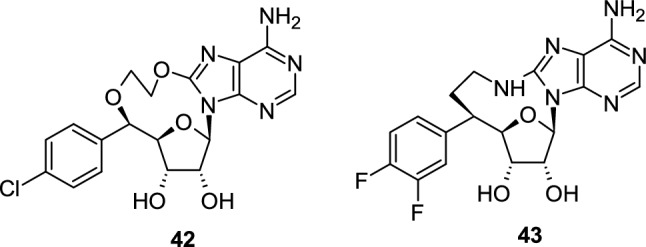
Cyclonucleosides **42**,**43** with PRMT5 inhibitory activity

Protein arginine methyltransferase 5 (PRMT5) functions as an epigenetic regulator responsible for the symmetrical dimethylation of histones H2AR3, H4R3, H3R2 and H3R8 in vivo, a common post-translational modification important in regulating chromatin function. Kawamura and co-workers reported the synthesis of 10- **42** and 9-membered **43** cyclonucleosides with PRMT5 inhibitory activity (Fig. 1) [27]. The co-crystal structure of PRMT5:MEP50 in complex with the cyclonucleoside **43** was obtained, and its binding mode in the SAM (S-adenosyl-L-methionine) binding pocket of PRMT5 was revealed.

**Scheme 11:**
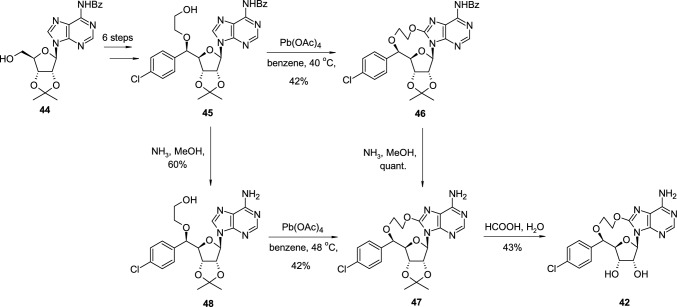
The synthesis of PRMT5 inhibitor **42** using Pb(OAc)_4_ as oxidative cyclisation agent

The synthesis of cyclonucleoside **42** started from isopropylidene-protected *N*-benzoyladenosine **44**, transformed in the six steps into intermediate **45**, where the C′5 position of the sugar ring was modified with the 5-chlorophenyl group and OH5′ was modified with hydroxyethyl group (Scheme 11). The cyclization step leading to the protected cyclonucleoside **46** was performed with lead tetraacetate (LTA) in warm benzene followed by the removal of the benzyloxy group with ammonia solution resulting in isopropylidene-protected cyclonucleoside **47**. Alternatively, intermediate **45** was deprotected with ammonia solution to **48**, and subjected to cyclisation with LTA. In the final step, the isopropylidene group was removed in acidic conditions with formic acid in water which led to the final cyclonucleoside **42**.

**Scheme 12:**
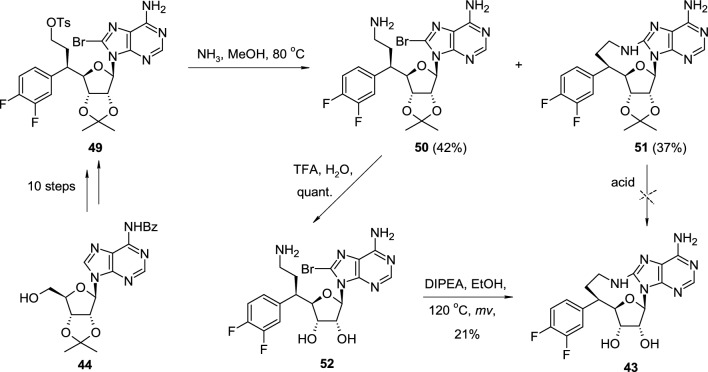
The synthesis of PRMT5 inhibitor **43** through intramolecular nucleophilic attack of primary aliphatic amine group on 8-bromoadenine ring

The synthesis of cyclonucleoside **43** started from isopropylidene-protected *N*-benzoyladenosine **44**, transformed in the ten steps into intermediate **49** possessing tosyl group in the side chain (Scheme 12). The treatment of **49** with methanolic ammonia led to the two products: the open-chain derivative **50** resulted from nucleophilic substitution of tosylate with ammonia and cyclonucleoside **51** resulted from a subsequent nucleophilic attack of an amine group on bromine-substituted purine ring. All attempts for the removal of the isopropylidene group in **51** were accompanied by the destruction of the aminoethylene bridge, which proved to be sensitive to acidic conditions. Consequently, open-chain intermediate **50** was first deprotected in acidic conditions to free nucleoside **52**, then transformed into cyclonucleoside **43** by heating with diisopropylethylamine (DIPEA) in ethanol under microwave conditions.

### The cyclisation approaches of acyclic purine nucleosides

**Scheme 13:**
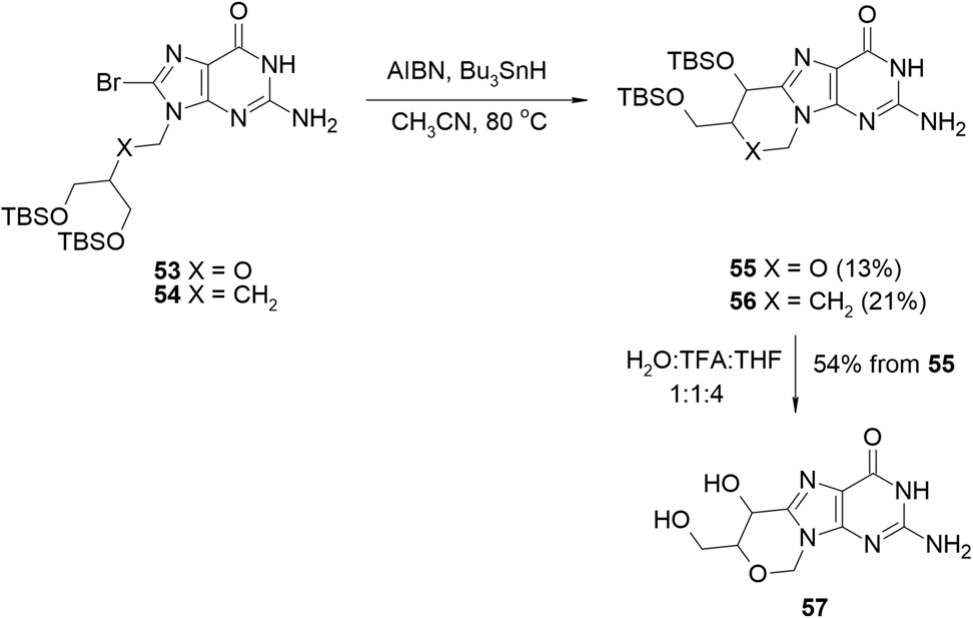
The radical cyclisation of TBS-protected Ganciclovir and Penciclovir **53**,**54** using AIBN/Bu_3_SnH system leading to C-bridged cyclonucleosides **55**,**56**

The acyclic purine nucleosides were also applied to cyclisation reactions resulting in bridged acyclonucleosides. Alvarenga, in her Ph.D. thesis published online, reported two methods of cyclisation of Ganciclovir and Penciclovir, the antiviral drugs [28]. In the first radical approach (Scheme 13), *tert*-butyldimethylsilyl (TBS) protected 8-bromo-Ganciclovir (**53**) and 9-bromo-Penciclovir (**54**) were heated with AIBN and tri(*n*-butyl)tin hydride in acetonitrile, which led to the *C*-bridged derivatives **55** and **56**, isolated from the reaction mixture with low yields (13% and 21%, respectively). Nucleosides **55** and **56** were then subjected to deprotection reaction in the presence of TFA, but only Ganciclovir cyclonucleoside **57** was isolated in the pure form with 54% yield.

**Scheme 14:**
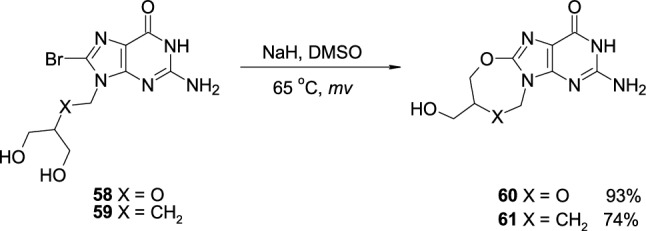
The synthesis of *O*-bridged analogues of Ganciclovir and Penciclovir **60**,**61** through nucleophilic attack of OH group on 8-bromoguanine ring

In the second approach [28], deprotected 8-bromo-Ganciclovir (**58**) and 8-bromo-Penciclovir (**59**) were heated in the presence of NaH in DMSO under the microwave conditions, which resulted in the formation of *O*-bridged derivatives **60** and **61**, isolated from the reaction mixture with high yields (93% and 74%, respectively) (Scheme 14).

**Scheme 15:**
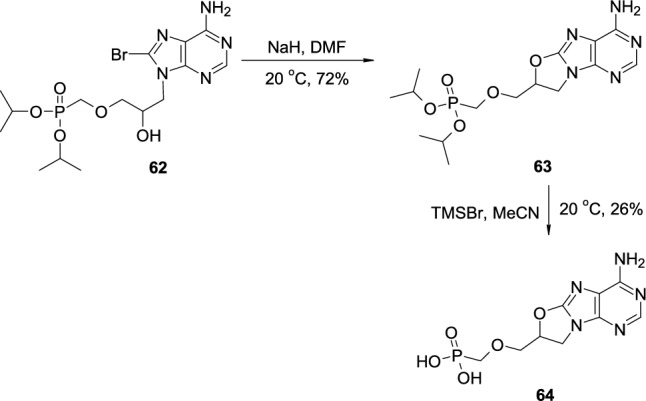
The synthesis of bridged acyclic nucleoside phosphonate ester **63** through nucleophilic attack of OH group on 8-bromoadenine ring

A similar approach was used by Janeba to synthesize bridged acyclic nucleoside phosphonate from *iso*-HPMPA [29] (Scheme 15). 8-Bromo-*iso*-HPMPA diisopropyl phosphonate **62** was treated with sodium hydride in DMF at room temperature, which led to the formation of bridged, acyclic, protected phosphonate ester **63**. Subsequent treatment with trimethylsilyl bromide led to the deprotection of the phosphonate group and the formation of *O*-bridged *iso*-HPMPA **64**.

**Scheme 16:**
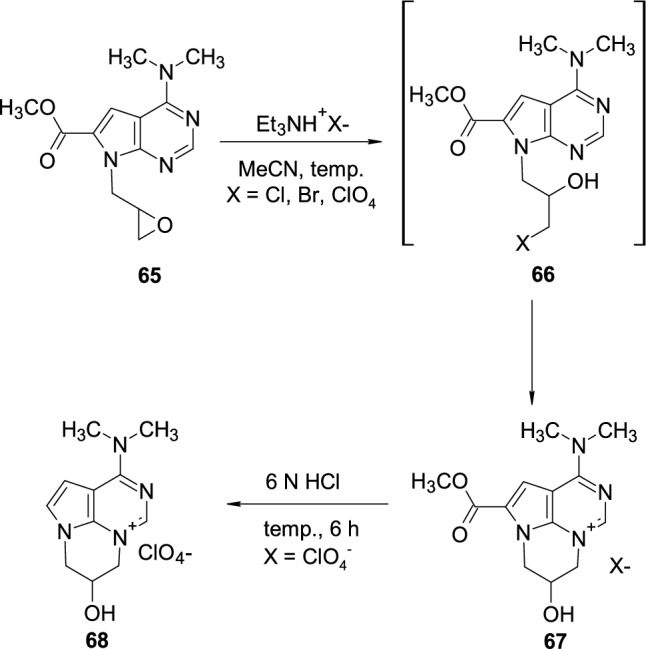
The synthesis of 7-deazapurine inner salts **67** through intramolecular attack of the halogen on *N*3 atom of the 7-deazapurine ring

Muzychka, during research on acyclic 7-deazapurine nucleoside analogues, observed the formation of cyclic salts resulting from the intramolecular attack of the halogen in the side chain on *N*3 atom of the 7-deazapurine ring [30, 31] (Scheme 16). 7-Deazapurine **65**, possessing an oxirane ring in the side chain, was heating in acetonitrile in the presence of triethylamine salts, which resulted in the opening of the oxirane ring and formation of intermediate **66** with subsequent intramolecular alkylation to inner salt **67**. 9-Deazapurine perchlorate salt **67**, when heated with hydrochloric acid, underwent ester hydrolysis followed by decarboxylation to product **68**.

### The synthesis of cyclonucleosides with non-canonical bases

**Scheme 17:**
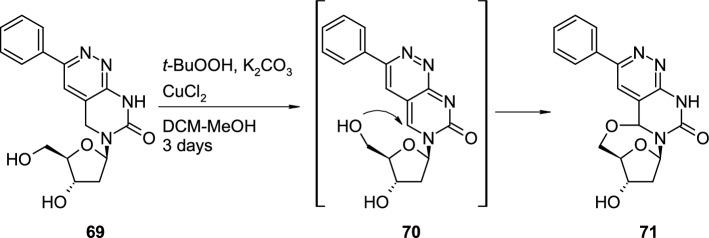
The radical approach to synthesis of 5,8-dihydropyrimido[4,5-*c*]pyridazin-7(6*H*)-one 6,5′-cyclonucleoside **71** using TBHP/CuCl_2_/K_2_CO_3_ system

Mei and co-workers synthesized 3-phenyl-5,8-dihydropyrimido[4,5-*c*]pyridazin-7(6*H*)-one 2′-deoxyriboside **69** to perform oxidative dehydrogenation leading to 3-phenylpyrimido[4,5-*c*]pyridazin-7(6*H*)-one 2′-deoxyriboside **70** [32]. The authors applied *tert*-butyl hydroperoxide (TBHP) as oxidant, catalytic amounts of CuCl_2_ and potassium carbonate (Scheme 17), but instead of 2′-deoxyriboside **70**, they identified 5,8-dihydropyrimido[4,5-*c*]pyridazin-7(6*H*)-one 6,5′-cyclonucleoside **71**, obtained in 55% yield, as the main product and nucleoside **70** was not detected in the reaction mixture.

**Scheme 18:**
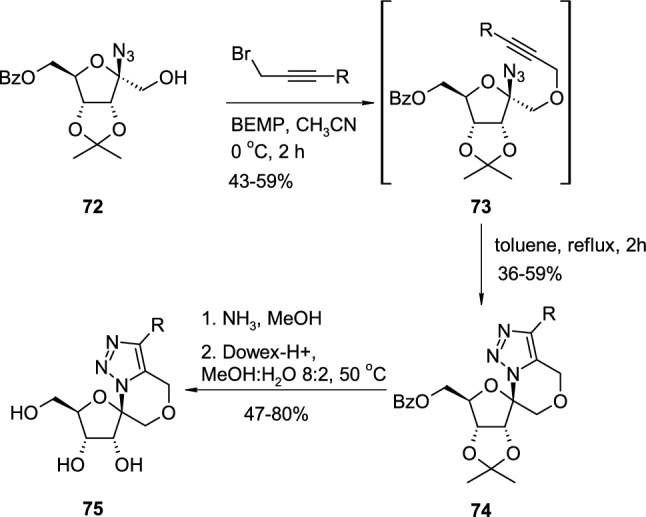
The synthesis of antiviral triazole spironucleosides **75** through tandem alkylation-azide ′click′intramolecular cycloaddition

An example of tandem alkylation-azide “click” intramolecular cycloaddition in the synthesis of cyclonucleoside derivatives was described by Dell′Isola and co-workers, who developed the spirocyclic [1,2,3]-triazolooxazine nucleosides as potential antiviral agents [33]. The sugar derivative **72** was subjected to the alkylation reaction with a range of propargyl bromides in the presence of BEMP (2-*tert*-butylimino-2-diethylamino-1,3-dimethylperhydro-1,3,2-diazaphosphorine) as a base to give the crude propargylic ether intermediates **73** (Scheme 18). Intermediate ethers **73** underwent efficient copper-free, thermal intramolecular 1,3-dipolar cycloaddition upon heating in toluene for 24 h, which resulted in the novel, protected spironucleosides **74**, which could be treated as cyclonucleosides bearing substituted triazole nucleosidic base. Nucleosides **74** was then subjected to a double deprotection reaction and treated with methanolic ammonia for the removal of the Bz group followed by treatment with Dowex-H + for the removal of the isopropylidene group. The final products **75** were tested for antiviral activity against coronaviruses using animal model coronavirus MHV (Mouse Hepatitis Coronavirus) grown in 17Cl-1 cells. One derivative substituted with a 4-chlorophenyl group (R = 4-ClC_6_H_4_) exhibited moderate antiviral activity against the MHV virus (EC_50_ = 36 μM) without noticeable cytotoxicity (CC_50_ > 2000 μM, SI > 56).

## The application of cyclonucleosides in the modification of nucleosides

The cyclonucleosides are widely used as a convenient synthetic tool for the modification of nucleosides and already found many applications in the synthesis of unique nucleoside analogues modified in sugar ring or heterocyclic base.

### Modification of heterocyclic base

**Scheme 19:**
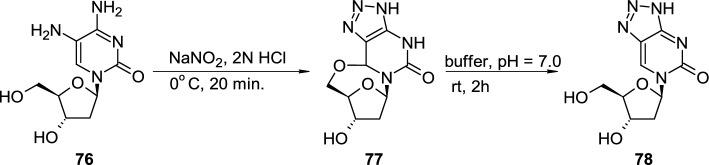
The synthesis of triazolopyrimidine 2′-deoxyriboside **78** through intermediate 6,5′-*O*-cyclonucleoside **77**

Mei reported the diazotization reaction of 5-amino-2′-deoxycytidine (**76**), treated with sodium nitrite under acidic conditions of 2 N HCl (Scheme 19) [32]. The only reaction product **77** had the 6,5′-*O*-cyclonucleoside structure, similar to structure **71**, and the open-chain product **78** was not initially detected in the reaction mixture. The author observed, that in contrast to cyclonucleoside **71**, relatively stable in aqueous conditions at neutral pH, 6,5′-*O*-cyclonucleoside **77** undergoes fast hydrolysis and conversion to triazolopyrimidine 2′-deoxyriboside **78**.

**Scheme 20:**
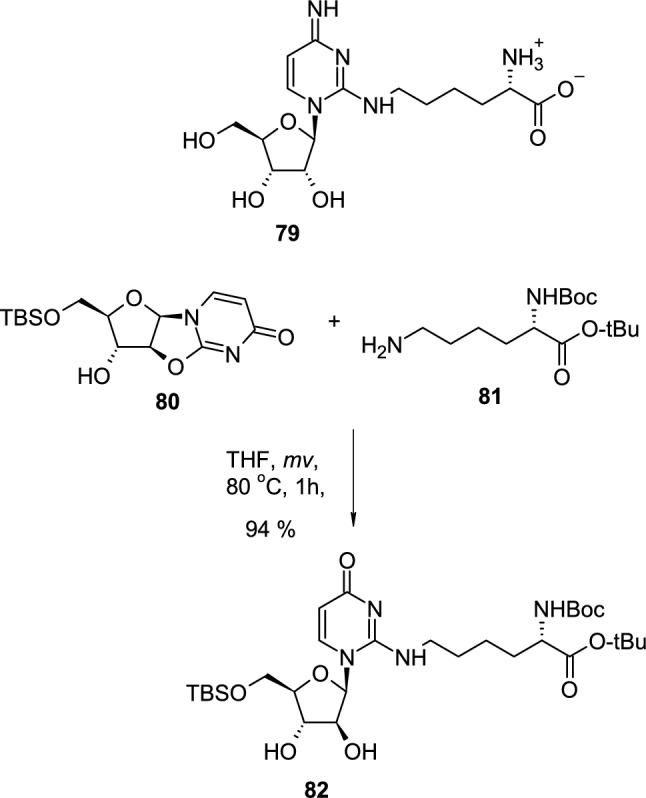
The synthesis of Lysidine analogue **82** through nucleophilic, intermolecular attack of aliphatic primary amine **81** on 2,2′-*O*-cyclo linkage of **80**

The recent examples of modifications of the heterocyclic base include improved microwave-assisted synthesis of isocytosine arabinosides [34]. The new approach allowed the reduction of reaction time to improve yield, and to perform the synthesis in the gram scale and was used for the synthesis of analogue of tRNA base—Lysidine (**79**). Thus 5′-*O-*TBS-2,2′-*O*-anhydrouridine (**80**) was heated with *N*^1^-Boc-lysine *t*-butyl ester (**81)**, under the microwave conditions, which led to the opening of the 2,2′-*O*-linkage and formation of isocytosine arabinoside **82** (Scheme 20).

### The synthesis of pyrimidine nucleosides with bi- and tricyclic sugar scaffolds

**Scheme 21:**
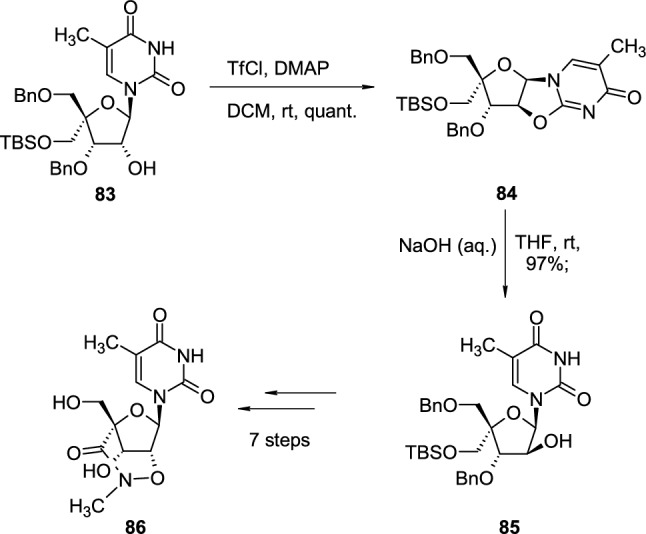
The synthesis of pyrimidine nucleoside **86** with bicyclic, perhydro-1,2-oxazin-3-one containing scaffold, through 2,2′-*O*-cyclonucleoside intermediate** 84**

Cyclonucleosides are widely used for structural modifications of sugar rings in nucleoside synthesis, including the preparation of bi- and tricyclic sugar scaffolds. In the synthesis of bridged nucleic acids [35], TBS- and Bn-protected pyrimidine nucleoside **83** was treated with trifluoromethanesulfonyl chloride (TfCl) in the presence of 4-(dimethylamino)pyridine (DMAP) in room temperature, which led to quantitative transformation to 2,2′-*O*-cyclonucleoside **84** (Scheme 21). Basic hydrolysis of 2,2′-*O*-cyclo linkage with sodium hydroxide led to intermediate nucleoside **85**, which was subjected to further structural transformations to obtain bicyclic nucleoside **86**, possessing a perhydro-1,2-oxazine-3-one ring.

**Scheme 22:**
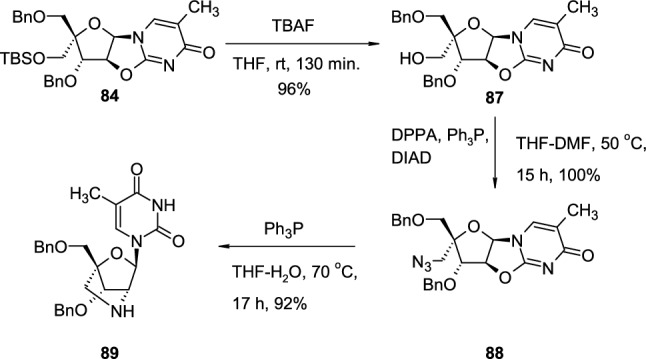
The synthesis of pyrimidine nucleoside **89** with bicyclic sugar scaffold through intramolecular attack of amine group on 2,2′-*O*-cyclo linkage

Protected cyclonucleoside **84** is a convenient intermediate for the synthesis of various nucleosides with bicyclic or tricyclic sugars, suitable building blocks for the development of novel locked nucleic acids (LNAs) [36] (Scheme 22). TBS-protected cyclonucleoside **84**, deprotected with TBAF in THF to intermediate **87**, is treated with diphenylphosphoryl azide (DPPA), in the presence of triphenylphosphine and diisopropyl azodicarboxylate (DIAD) which led to 5′-azidomethyl cyclonucleoside **88**. The reduction of the azide group to the amine group with triphenylphosphine, followed by the subsequent nucleophilic attack of the primary, aliphatic amine on 2,2′-*O*-cyclo linkage resulted in Bn-protected bicyclic nucleoside **89**.

**Scheme 23:**
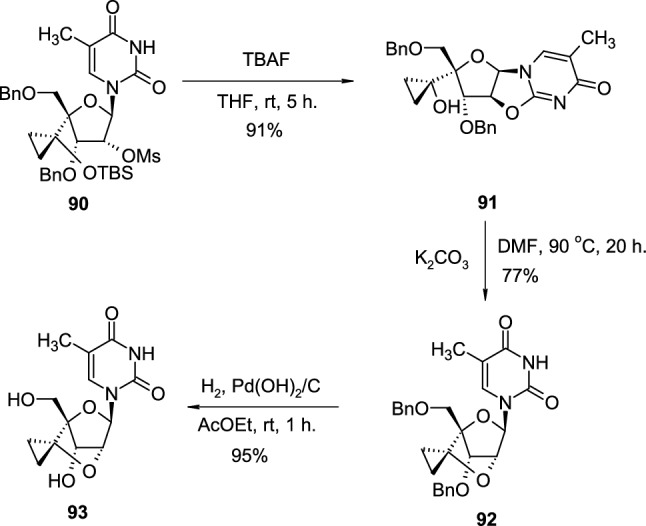
The synthesis of pyrimidine nucleoside **93** with tricyclic, spiro-cyclopropane containing sugar scaffold through intramolecular attack of OH group on 2,2′-*O*-cyclo linkage

In the another example of application of pyrimidine 2,2′-*O*-cyclonucleoside in the synthesis of building blocks for LNAs, Yamaguchi reported the synthesis and properties of 2′-*O*,4′-*C*-spirocyclopropylene bridged nucleic acids (scpBNA), an analogue of 2′,4′-BNA/LNA bearing a cyclopropane ring [37]. For this purpose, sugar-modified, 2′-OMs pyrimidine nucleoside **90** was transformed to 2,2′-*O*-cyclonucleoside **91** using TBAF (Scheme 23). In the presence of potassium carbonate, at elevated temperature, 2,2′-*O*-cyclonucleoside **91** was rearranged to benzyl-protected intermediate **92**, bearing tricyclic sugar scaffold. Finally, deprotection to final nucleoside **93** was performed by hydrogenation reaction, giving the requested product **93** with a high yield.

**Scheme 24:**
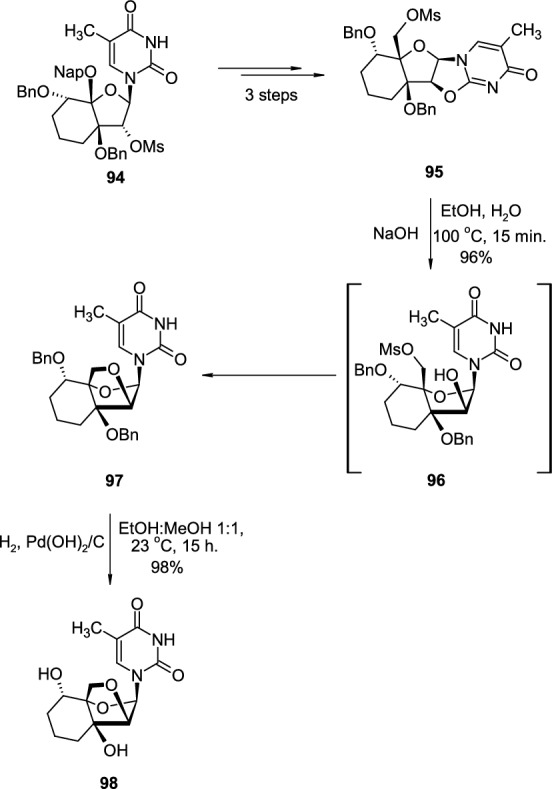
The synthesis of thymidine analogue **98** from 2,2′-*O*-cyclonucleoside intermediate **95**, through intramolecular attack of 2′-OH group on 5′-OTs group

A constrained tricyclic thymidine analogue was also reported by Hanessian (Scheme 24) [38]. Bicyclic, Nap/Bn-protected pyrimidine nucleoside analogue **94** was transformed in the three steps into 2,2′-*O*-cyclonucleoside **95**. The hydrolysis of ether linkage in **95** with NaOH led to intermediate **96**, which immediately underwent cyclisation through nucleophilic attack of 2′-OH group on 5′-OMs group, giving protected pyrimidine nucleoside **97** bearing tricyclic sugar scaffold. The deprotection step, removal of benzyl groups was performed by hydrogenation reaction, resulted in final product **98**.

### Reactions of *O*-bridged pyrimidine cyclonucleosides with nucleophilic reagents

The successful synthesis of pyrimidine nucleosides with bi- and tricyclic sugar scaffolds, presented in the previous paragraph, was mainly based on intramolecular attacks of nucleophilic groups such as OH or NH_2_ on 2,2′-*O*-cyclo linkage of appropriate pyrimidine cyclonucleoside. Similarly, the intermolecular attack of various nucleophilic reagents on 2,2′-*O*-cyclo or 2,3′-*O*-cyclo linkages could lead to valuable, sugar-modified pyrimidine nucleoside derivatives.

**Scheme 25:**
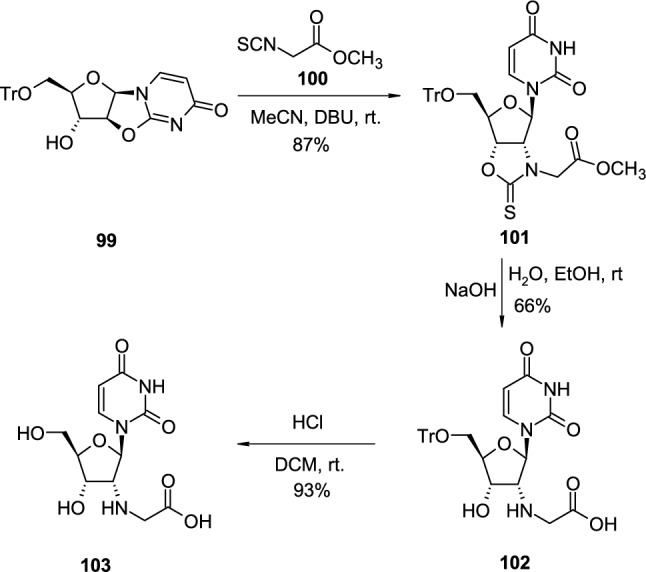
The synthesis of C′2 aminoacid-substituted pyrimidine nucleoside **103** through nucleophilic attack of isothiocyanate group on pyrimidine 2,2′-*O*-cyclonucleoside **99**

The pyrimidine 2,2′-*O*-cyclonucleoside intermediates were used to introduce diamine or aminoacid functional groups to C'2 position of ribonucleosides [39] (Scheme 25). In the representative example, 5′-*O*-trityl-2,2′-*O*-cyclouridine (**99**) was treated with methyl 2-isothiocyanatoacetate (**100**) in the presence of DBU, which resulted in the formation of bicyclic nucleoside **101** with a new tetrahydrofuro[3,4-*d*]-oxazole-2(3*H*)-thione heterocyclic ring. Treatment of **101** with sodium hydroxide led to concomitant cleavage of the oxazole ring and hydrolysis of the ester group giving trityl-protected nucleoside **102**. The removal of the trityl group with hydrochloric acid resulted in deprotected 2′-amino-2′-deoxyriboside **103**.

**Scheme 26:**
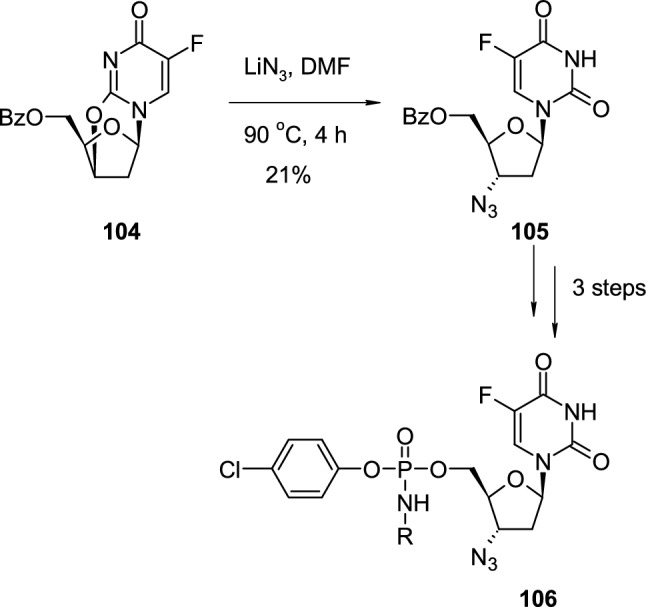
Synthesis of anticancer 3′-azido-2′,3′-dideoxy-5-fluorouridine phosphoramidates **106** through nucleophilic attack of N_3_.^−^ on 2′-deoxy- 2,3′-*O*-cyclonucleoside **104**

Pyrimidine cyclonucleosides are convenient intermediates for the introduction of azide N_3_ group to the sugar ring. Lewandowska reported the synthesis of the series of 3′-azido-2′,3′-dideoxy-5-fluorouridine phosphoramidates and evaluated their anticancer activity [40] (Scheme 26). The cyclonucleoside, 5′-*O*-benzoyl-2′-deoxy-5-fluoro-2,3′-*O*-cyclouridine (**104**), was heated with lithium azide in DMF for 4 h, which resulted in the formation of 3′-azido-5′-*O*-benzoyl-2′,3′-dideoxy-5-fluorouridine (**105**) with low yield. All attempts to increase the yield of this reaction failed, and the obtained derivative **105** was then converted into the corresponding 3′-azido-2′,3′-dideoxy-5-fluorouridine phosphoramidates **106**. Phosphoramidates **106** were tested for their anticancer activity on three human cancer cell lines: cervical (HeLa), oral (KB) and breast (MCF-7), showing, in some cases, a higher cytotoxic effect than reference 5-fluoro-2′-deoxyuridine (FdU), 3′-azido-2′,3′-dideoxy-5-fluorouridine (AddFU) and Cytarabine.

**Scheme 27:**
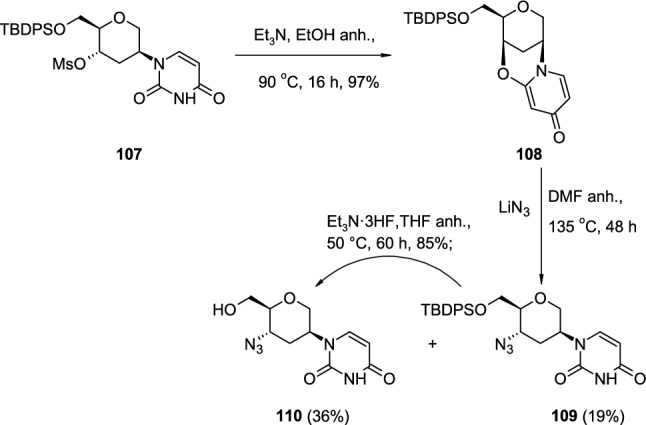
Introduction of N_3_ group to 4′position of hexitol nucleoside **109** through nucleophilic attack of N_3_.^−^ on pyrimidine 2,4′-*O*-cyclonucleoside **108**

De reported the synthesis of protected 4′-aminohexitol nucleoside building blocks suitable for oligonucleotide synthesis, where 4′-OH mesylated nucleoside **107** underwent basic cyclisation to 2,4′-*O*-cyclonucleoside **108** in the presence of triethylamine in refluxing ethanol [41] (Scheme 27). The nucleophilic attack of azide anion on 2,4′-*O*-cyclo linkage leading to 4′-azido substituted nucleoside **109** was partially accompanied by the removal of the TBDPS protecting group resulting in nucleoside **110**. Protected 4′-azido substituted nucleoside **109** was also subjected to a deprotection reaction leading to **110** by treatment with Et_3_N*3HF reagent in dry THF.

**Scheme 28:**
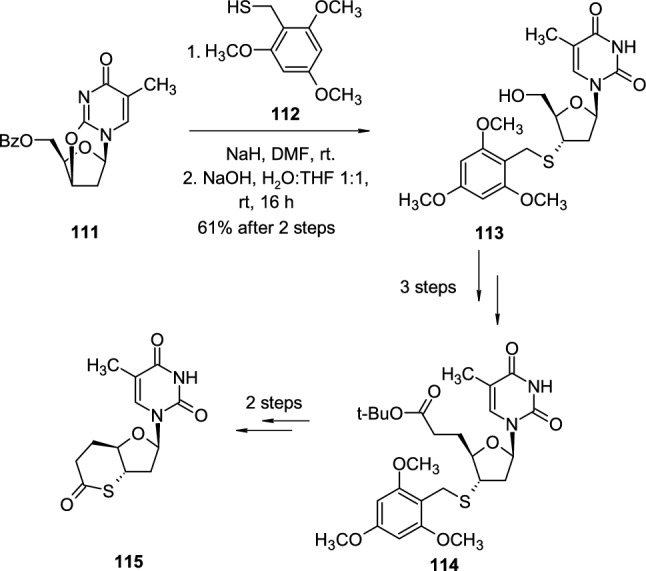
Synthesis of 3′-deoxy-3′-thiothymidine **113** through nucleophilic attack of thiolate on 2,3′-*O*-cyclothymidine **111**

Except for the introduction of azide and amine groups, cyclonucleosides could be convenient intermediates to introduce thiol groups to the sugar ring [42] (Scheme 28). 2,3′-*O*-cyclo-5′-*O*-benzoylthymidine (**111**) was treated with 2,4,6-trimethoxybenzyl mercaptan (**112**), in the presence of sodium hydride, which led to the nucleophilic attack of thiolate on 2,3′-*O*-cyclo linkage. As partial deprotection of the 5′OH group occurred during the substitution step, the reaction mixture was directly subjected to full deprotection, the remaining benzoyl group was removed with a sodium hydroxide solution resulting in 3′-thiobenzyl intermediate **113** obtained after two steps with 61% yield. Nucleoside **113** was then transformed in 3 subsequent steps into the key intermediate **114**, which after two next steps gave bicyclic thiolactone nucleoside **115**. The corresponding 2′-deoxycytidine analogue of **115** was also obtained using a slightly modified procedure.

**Scheme 29:**
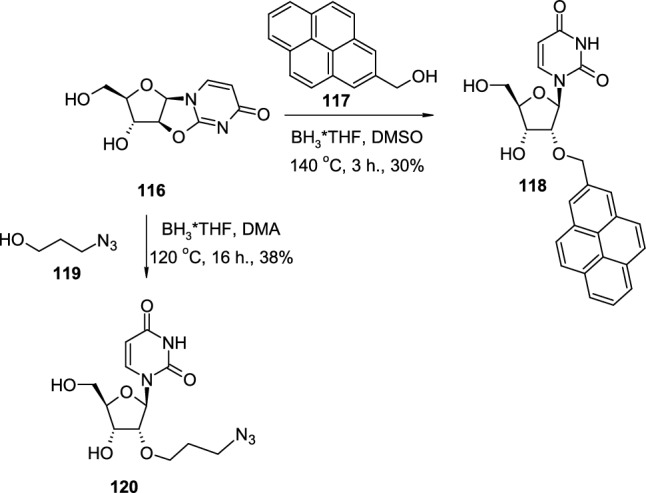
The synthesis of 2′-alkoxy derivatives **118**,**120** of uridine from 2,2′-*O*-cyclouridine **113** and alcohols **117**,**119** in the presence of BH_3_*THF

The use of pyrimidine 2,2′-*O*-cyclonucleosides also enables the introduction of 2′-alkoxy groups to the nucleoside structure (Scheme 29). In the first example, unprotected 2,2′-*O*-cyclouridine (**116**) is treated with 2-pyrenemethanol (**117**) in the presence of borane-THF complex at elevated temperatures, which leads to the *O*′2-(2-pyrenemethyl) derivative **118** [43]. In the another example, 2,2′-*O*-cyclouridine (**116**) underwent reaction with 3-azidopropanol (**119**) under similar conditions, which results in the formation of *O*′2-(3-azidopropyl) derivative **120** [44].

### Various applications

**Scheme 30:**
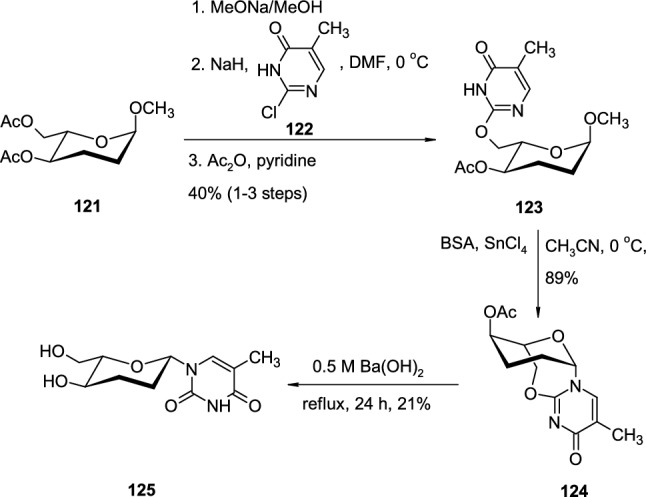
Synthesis of *β*-L-*erythro*-hexopyranosyl nucleoside **125** via 2,6′-*O*-cyclonucleoside intermediate **124**

The interesting application of 2,6′-*O*-cyclo linkage in the synthesis of pyrimidine *β*-L-*erythro*-hexopyranosyl nucleosides, suitable for the development of L-Homo-DNA, was presented by D′Alonzo [45] (Scheme 30). Acetyl-protected sugar **121** was subjected to a sequence of reactions including deprotection of the hydroxyl group, coupling with the sodium salt of heterocyclic base **122** (intermolecular *O*-glycosylation) followed by protection of hydroxyl groups with acetic anhydride in pyridine. These transformations led to the formation of *O*-glycosidic product **123**, where sugar and heterocyclic base are linked by an ether linkage. *O*-Glycosidic intermediate **120** was then subjected to intramolecular *N*-glycosidation in the presence of bis(trimethylsilyl)acetamide (BSA) and SnCl_4_, which resulted in the formation of 2,6′-*O*-cyclonucleoside **124**. The authors observed that hydrolysis of the *O*-glycosidic bond in **124** occurred under treatment with hydroxide ions after prolonged reaction times (24 h at reflux). The last step was also accompanied by deprotection of the hydroxy group giving the final product **125** in low yield.

**Scheme 31:**
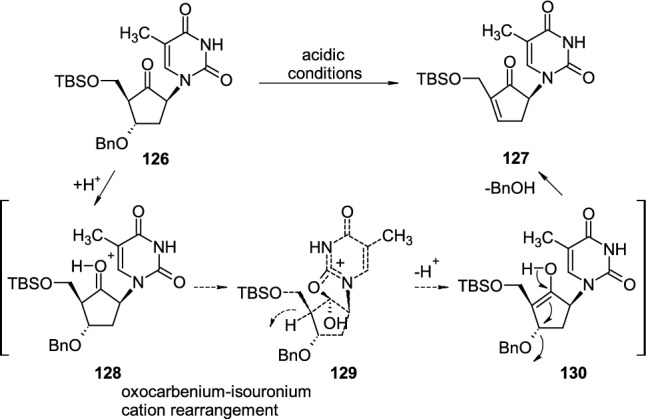
Formation of unsaturated carbocyclic ketonucleoside **127** from **126** through cyclonucleoside isouronium intermediate **129**

Belostotskii reported the synthesis of carbocyclic ketonucleoside **126**, which loses the benzyloxy group in acidic conditions and undergoes fast transformation to unsaturated nucleoside **127** [46] (Scheme 31). The authors proposed the mechanism of elimination through intermediate forms **128**–**130**, which include the protonation of the keto group and rearrangement of oxocarbenium **128** to cyclonucleoside isouronium kation **129** followed by elimination of benzyl alcohol from **130** leading to product **127**.

**Scheme 32:**
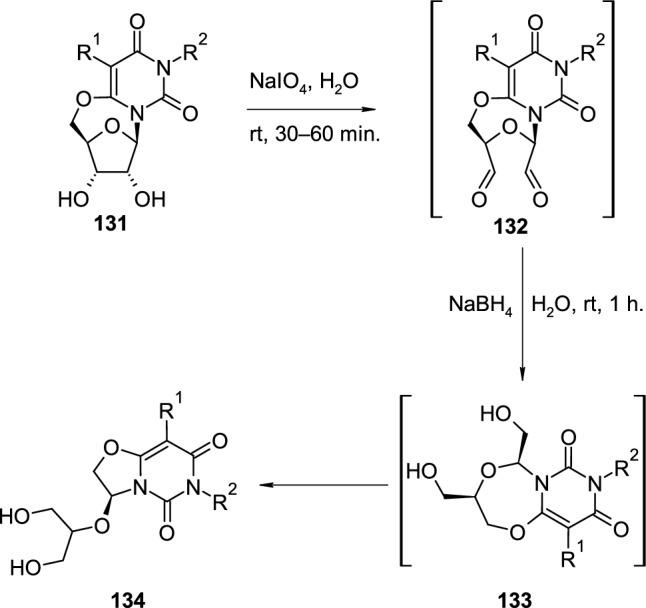
Transformation of 6,5′-*O*-cyclouridines **131** to 6*H*-oxazolo[3,2-*f*]pyrimidine-5,7-diones **134** via oxidation–reduction-rearrangement cascade process

Cyclonucleoside are not only suitable for modifications of sugar rings or heterocyclic bases of nucleoside analogues, but also could be transformed into other, fused heterocyclic systems. Mieczkowski et al. reported an interesting transformation of 6,5′-*O*-cyclouridines to 6*H*-oxazolo[3,2-*f*]pyrimidine-5,7-diones [47] (Scheme 32). The developed method started from deprotected 6,5′-*O*-cyclouridine **131**, treated in the first step with sodium periodate in water which resulted in the oxidation of the ribose *cis-*diol group and the formation of dialdehyde **132**. The treatment of dialdehyde **132** with sodium borohydride caused the reduction of aldehyde groups to hydroxyl groups leading to a rather unstable 7-membered intermediate **133**. The reduction process is accompanied by fast subsequent ring contraction and rearrangement of the dioxazepine ring in **133** to five-membered oxazole ring observed in the final product **134**. The molecular structure of the final compounds was confirmed by two crystal structures.

**Fig. 2:**
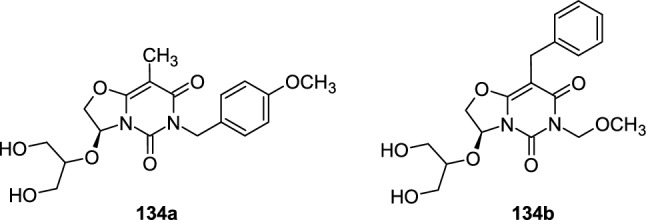
The examples of 6*H*-oxazolo[3,2-*f*]pyrimidine-5,7-diones **134a,b**

Products **134** could also be treated as *O*-bridged, acyclic pyrimidine nucleoside analogues or acyclic purine nucleoside analogues. The representative structures **134a**,**b** are shown in Fig. 2. The 11 final products were tested for their cytotoxic activity on six cancer and one non-cancer cell line but exhibited relatively weak cytotoxicity.

## Conclusion

Cyclonucleosides are valuable nucleoside analogues which could be synthesized by various methods leading to a diverse range of heterogeneous groups of compounds varied from each other by a heterocyclic base, sugar ring and type of linkage (length, structure and attachment to the rings). Their rigid, fixed structure could determine their biological properties and could be a starting point for the development of enzyme inhibitors (PRMT5) and molecular tools for biochemistry. Cyclonucleosides could exhibit anticancer, antiviral or antibacterial activity and could be introduced to oligonucleotide chains. Cyclonucleosides with labile linkages, susceptible to attack of nucleophilic reagents, could be valuable intermediates for the synthesis of nucleoside analogues, enabling modifications of heterocyclic base (i.e. synthesis of isocytidine nucleosides), or sugar ring (introduction of azide, amine, thiol or alkoxy groups). They found applications in the synthesis of nucleosides with bicyclic sugars, suitable for the development of locked nucleic acids. Cyclonucleoside intermediates were used to perform the intramolecular *N*-glycosylation and were transformed into other heterocyclic systems (oxazolopyrimidines). Consequently, cyclonucleosides are an important, and valuable group of nucleosides which could be applied in both basic (new synthetic tools) and applied (inhibitors, biocides, biochemical tools) chemical sciences.

## Abbreviations

1,6-HAT cyclisation: 1,6-Hydrogen atom transfer cyclization
AddFU: 3′-Azido-2′,3′-dideoxy-5-fluorouridine
AdoHcy: *S*-Adenosylhomocysteine, SAH
AIBN: Azobisisobutyronitrile
BEMP: 2-*tert*-Butylimino-2-diethylamino-1,3-dimethylperhydro-1,3,2-diazaphosphorine
BSA: Bis(trimethylsilyl)acetamide
DAST: Diethylaminosulfur trifluoride
DCM: Dichloromethane
DBU: 1,8-Diazabicyclo[5.4.0]undec-7-ene
DIAD: Diisopropyl azodicarboxylate
DIPEA: Diisopropylethylamine
DMAP: 4-(Dimethylamino)pyridine
DPPA: Diphenylphosphoryl azide
DTBP: Di-*tert*-butyl peroxide
DTBMP: 2,6-Di-*tert*-butyl-4-methylpyridine
FdU: 5-Fluoro-2′-deoxyuridine
HMDS: Hexamethyldisilazane
HPMPA: [(2*S*)-1-(6-Aminopurin-9-yl)-3-hydroxypropan-2-yl]oxymethylphosphonic acid
LNAs: Locked nucleic acids
LTA: Lead tetraacetate
MEP50: Methylosome protein 50
MHV: Mouse hepatitis coronavirus
MTA: 5′-Methylthiodenosine
MTA/AdoHcy nucleosidase: 5′-Methyl-thioadenosine/*S*-adenosyl-homocysteine nucleosidase
PRMT5: Protein arginine methyltransferase
SAH: *S*-Adenosylhomocysteine
SAM: *S*-Adenosyl-L-methionine
TBAF: Tetra-*n*-butylammonium fluoride
TBHP: *tert*-Butylhydroperoxide
TBDPS: *tert*-Butyldiphenylsilyl
TBS: *tert*-Butyldimethylsilyl
TCBQ: Tetrachloro-1,4-benzoquinone
TfCl: Trifluoromethanesulfonyl chloride
TIPS: Triisopropylsilyl
TMSOTf: Trimethylsilyl triflate
TMS_3_SiH: Tris(trimethylsilyl)silane
